# Neuroinflammatory responses in diabetic retinopathy

**DOI:** 10.1186/s12974-015-0368-7

**Published:** 2015-08-07

**Authors:** Ying Yu, Hui Chen, Shao Bo Su

**Affiliations:** State Key Laboratory of Ophthalmology, Zhongshan Ophthalmic Center, Sun Yat-sen University, 54 S Xianlie Road, Guangzhou, 510060 China; Eye Institute, Affiliated Hospital of Nantong University, Nantong, 226001 China

**Keywords:** Neural retina, Neuroinflammation, Diabetic retinopathy, Cytokine

## Abstract

Diabetic retinopathy (DR) is a common complication of diabetes and has been recognized as a vascular dysfunction leading to blindness in working-age adults. It becomes increasingly clear that neural cells in retina play an important role in the pathogenesis of DR. Neural retina located at the back of the eye is part of the brain and a representative of the central nervous system. The neurosensory deficits seen in DR are related to inflammation and occur prior to the clinically identifiable vascular complications. The neural deficits are associated with abnormal reactions of retina glial cells and neurons in response to hyperglycemia. Improper activation of the innate immune system may also be an important contributor to the pathophysiology of DR. Therefore, DR manifests characteristics of both vasculopathy and chronic neuroinflammatory diseases. In this article, we attempt to provide an overview of the current understanding of inflammation in neural retina abnormalities in diabetes. Inhibition of neuroinflammation may represent a novel therapeutic strategy to the prevention of the progression of DR.

## Introduction

Diabetic retinopathy (DR) is a common complication of diabetes and a leading cause of legal blindness in working-age adults in the world [1, 2]. According to the report of World Health Organization (WHO), the prevalence of DR is expected to increase and the number of people at the risk of vision loss is predicted to double by the year 2030 [3]. DR is staged into several levels of severity, including mild, moderate, and severe nonproliferative DR (NPDR), followed by an advanced proliferative DR (PDR), as defined by the presence of retinal neovascularization [4]. In PDR, proliferative neovasculature causes severe complications, such as vitreous hemorrhage, retinal scars, and tractional retinal detachment, all of which may lead to irreversible vision loss.

**Fig. 1:**
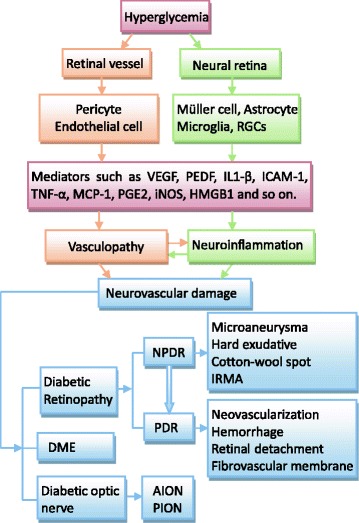
Clinicopathologic characteristics of the neural retina in diabetes. DR manifests characteristics of both vasculopathy and neuroinflammatory diseases. Neural retina including retina glial cells and neurons is involved in the neuroinflammatory responses of DR. *NPDR* nonproliferative diabetic retinopathy, *PDR* proliferative diabetic retinopathy, *RGCs* retinal ganglion cells, *IRMA* intraretinal microvascular anomalies, *DME* diabetic macular edema, *AION* anterior ischemic optic neuropathy, *PION* posterior ischemic optic neuropathy

The clinical evidence indicates that there is an increased capillary permeability and capillary occlusion in DR. Many DR studies in both clinic and animal models focused on vascular dysfunction, such as impaired endothelial cells, death of pericytes, thickening of retina capillary basement membrane, and altered tight junctions [5, 6]. However, retinal thickness, as measured by the retinal thickness analyzer, has been found to be abnormally diffused in the retina including the areas without clinically apparent retinopathy [7]. Microaneurysms, acellular capillary, and pericyte ghosts are more numerous in the temporal retina than in the nasal retina. However, the change in the thickness of retina capillary basement membrane is similar in all retina areas of retinopathy [8]. Both DR and diabetic nephropathy are considered as microvascular complications of diabetes. However, diabetic microvasculopathy may not explain the susceptibility of peripheral nerves or cerebral complications [9]. Thus, DR may not simply be a vasculopathy.

Recent studies revealed that electroretinogram (ERG) is defective in patients with diabetes who have no clinical retinopathy [10, 11]. The thickness of the nerve fiber layer in retinal superior polar quadrant was significantly reduced in patients with 15-year diabetic history, suggesting a loss of axons in this area [12]. In addition, functional changes in the earliest stages of human diabetic retinopathy were detected prior to the development of vascular dysfunction; therefore, the effect of hyperglycemia may be direct on the neural retina rather than secondary to the breakdown of the blood-retinal barrier [13]. Actually, neural retina located in the back of the eye is the evagination of the brain and a representation of the central nervous system (CNS). It is noted that chronic neurodegeneration is a critical cause of vision loss in DR [14]. The neurosensory deficits in DR occur prior to the clinically identifiable vascular complications [15]. Then, a major question is what links the neural responses of the retina, brain, and peripheral nerves and makes the neural tissues susceptible to hyperglycemia?

Epidemiologic studies have shown an association between the appearance of inflammatory biomarkers and the occurrence of type 2 diabetes mellitus (T2DM) and its complications [16]. Diabetics have increased serum levels of inflammatory markers, including C-reactive protein (CRP), interleukin-6 (IL-6), and tumor necrosis factor-alpha (TNF-α) [17]. There is increasing evidence that inflammatory processes play a considerable role in the pathogenesis of DR [18]. Leukostasis is particularly increased in the retinas of diabetic mice [19], while leukostasis in rats is associated with retinal endothelial cell injury and death [20]. There is also an association between high levels of proinflammatory cytokines and the development of diabetic retinopathy [21]. Although DR exhibits features of chronic neuroinflammation, the precise relationship between inflammatory alterations in DR and the loss of neural function is currently unknown.

In order to delineate inflammatory processes involved in DR, we will provide an overview of the current understanding of retinal neural abnormalities evoked by inflammation. Although treatment of vascular disorders in later stages of the disease may preserve vision in many DR patients, prevention of the onset of the disease or arresting its progression at early stage of chronic inflammation is highly desired.

## The retinal neural system

The neural retina is a highly specialized nervous tissue, a part of the brain. It is divided into nine layers. From developmental perspective, the retina is a heterocellular collection of interacting cellular systems assembled by three distinct neuron-like groups: 1, superset of rod and cone photoreceptors and bipolar cells [22]; 2, superset of amacrine cells, axonal cells, and ganglion cells (GCs) [23]; and 3, the gliaform cell phenotype and the superclass of horizontal cells. In addition, a complete vertebrate retina contains two traditional classes of glial cells: Müller’s cells and astrocytes. Furthermore, functional neuroanatomy contains not only the neuronal architecture for signal processing but also the synaptic connectivity, network topology, and signaling biophysics of retinal networks.

DR involves alterations of all retinal cellular elements, including vascular endothelial cells, pericytes, glial cells (macroglia/microglia), and neurons (photoreceptors, bipolar cells, amacrine cells, and ganglion cells), showing a diffused pathological process.

## Clinicopathologic and bioelectrical characteristics of the neural retina in diabetes

DR is characterized by a long period of clinical silence without significant signs and symptoms. However, by the time worsening vision is experienced, pathology may have been significantly advanced. Visual electrophysiology including ERG and visual-evoked potentials (VEPs) can reveal the earliest sign of impairment of retinal and optic nerve function both in diabetic human and model animals [24–26]. Oscillatory potentials (OPs) relating to amacrine cells, GCs and Müller cells [27], are considered as sensitive indicators of DR in diabolic patients and model rats [28–30]. The clinicopathologic and bioelectrical characteristics of retina in diabetes are shown in Table 1.

### Retinopathy in diabetes

NPDR stages in patients are determined by the number and severity of microaneurysms, dot-and-blot hemorrhages, hard exudates [31], cotton-wool spots, venous abnormalities, and intraretinal microvascular anomalies (IRMAs) [32]. PDR is a characteristic of neovascularization. The clinicopathologic features of the neural retina in NPDR and PDR are shown in Fig. 1. Clinically, there is a stage called “no apparent retinopathy”, which also can be labeled as NPDR grade 1. During this stage, there is no apparent morphological change. However, multifocal ERG (mfERG) technology reveals that functional changes are measurable in patients with diabetes without classic indicators of retinopathy [33, 34], suggesting that disfunction occurs before the appearance of morphological changes [35]. Local mfERG implicit times are significantly prolonged in the eyes of diabetic subjects without retinopathy [36]. In patients with type 1 diabetes of 3 months without retinopathy, VEP recordings show delayed P100 implicit time but with amplitudes similar to those of control subjects [37].

**Table 1:** Clinicopathologic and bioelectrical characteristics of retina in diabetes

Morphological features	Clinicopathologic features	Bioelectrical features	Model	References
No apparent retinopathy	–	mfERG: implicit times prolong	Human	[36, 37]
		VEP: P100 implicit time delay	Human	
Microaneurysma	Loss of pericytes		Rat	[5]
Hard exudative	Degeneration of photoreceptor and neuronal elements in the outer plexiform layer		Human	[31]
Cotton-wool spot	Microinfarct of the nerve fiber layer	PERG: amplitude reduce	Human	[25, 32]
IRMA	Shunt vessels and re-vascularize the hypoxic neuropile		Human	[32]
Neovascularization	Disrupt local basement membrane	OPs amplitude is correlated with the grade of DR	Human	[28, 39]
Hemorrhage			Human	[38]
Dot-and-blot	Hemorrhage in the inner nuclear layer			
Flame-shaped	Hemorrhage in the nerve fiber layer			
Globular	Hemorrhage in the middle neural retinal layer			
Confluent	Hemorrhage in all neural retinal layers			
Massive	Hemorrhage break through internal limiting membrane			
Retina detachment	Shrinkage of the fibroglial component			
Fibrovascular membrane	Composed of blood vessels, fibrous, glial matrix tissue, fibroblasts, and glial cells			
DME	Intracellular fluid collections in Müller cells, extracellular fluid in the outer plexiform and the inner nuclear layers	mfERG: P1 latency decrease	Human	[41, 44, 45]
		Macular OPs: reduced	Human	
Diabetic optic nerve	Vascular leakage and axonal edema in and around the optic nerve head	Increased VEP latency	Rat	[26, 47]

The neovascularization in PDR, which is initially intraretinal, usually breaks through the internal limiting membrane and lies between the membrane and the vitreous. Neovascularization is eventually accompanied by hemorrhage [38] and retinitis proliferation. Shrinkage of the fibroglial component often leads to neural retinal detachment. In mild background diabetic retinopathy, the reduced amplitudes of pattern electroretinogram (PERG) reveal the presence of cotton-wool spots and angiographic evidence of capillary nonperfusion [25], suggesting that PERG has certain advantages as a screening test when NPDR deteriorates to a PDR stage. Some study considers the amplitude of OPs as the prediction of the progression of eyes with NPDR or mild PDR to severe PDR, and the changes correlate with the grade of DR [28, 39]. The significantly reduced OP amplitudes indicate a high risk to develop proliferative diabetic retinopathy [28]. Moreover, the probability of regression curves based on lower OPs amplitudes, greater retinopathy severity, higher fluorescein leakage, and higher capillary nonperfusion can be used to support clinical decisions concerning the time to perform panretinal laser photocoagulation and how often to follow up [40].

### Diabetic macular edema

Clinically diabetic macular edema (DME) is the most important single cause of vision impairment in diabetic patients. Morphologic evidence suggests that macular edema may be caused by functional damage to the retinal vascular endothelium, resulting from intracellular fluid collection in Müller cells. Excessive swelling and rupture or death of Müller cells produce pockets of fluid and cell debris. Extracellular fluid mainly in the outer plexiform and the inner nuclear layers of the central part of the retina may also result in macular edema [41], causing similar changes in adjacent neurons. mfERG may evaluate the macular function with high sensitivity [42, 43]. The response of the positive wave (P1) in macular and paramacular areas tends to decrease in latency and increase in amplitude 3 months after vitrectomy of diabetic macular edema [44]. Macular OPs are reduced in diabetic maculopathy, leaving the a- and b-waves intact, suggesting macular OPs can also be a sensitive indicator to assess the macular function of DME [45].

### Diabetic optic neuropathy

Diabetes is a known risk factor for the development of ischemic optic neuropathy, particularly non-arteritic anterior ischemic optic neuropathy (NA-AION) and posterior ischemic optic neuropathy (PION) [46]. It is characterized by optic disc swelling caused by vascular leakage and axonal edema in and around the optic nerve head [47]. VEP supports the diagnosis of the AION, which causes optic disc edema in type-I diabetes [48]. Increased VEP latency is statistically correlated with the changes of the glucose level in the blood [26].

## The role of inflammatory mediators and adhesion molecules in the pathogenesis of DR

A considerable body of evidence from animal models and patients shows that DR is a chronic low-grade inflammatory disorder with participation of inflammatory mediators [49, 50]. Cytokines, chemokines, adhesion molecules, prostaglandins, and inflammatory cells including macrophages and neutrophils participate in a complex chain of events [41, 51, 52]. The inflammatory responses of specific cell types in DR are shown in Table 2.

**Table 2:** Retina cells involved in inflammatory responses in DR

Neural cell	Inflammatory molecule	Model	References
Müller cell	VEGF	Rat/mice	[76, 79]
	PEDF	Rat	[79]
	IL1-β	Rat/human	[83]
	TNF-α	rMC-1 cells	[78]
	MCP-1	rMC-1 cells	[78]
	β-catenin	Mice	[81]
	NO, COX2	Rat/rMC-1 cells	[86]
	PGE_2_, iNOS	Rat/rMC-1 cells	[86]
	RAGE	Rat/human Müller cell	[87]
	S100B	Rat/human Müller cell	[87]
	IL-6	Human Müller cell	[74]
Astrocyte	COX-2	Human	[102]
	IL-1β	Rat	[103]
Microglia	TNF-α	Rat	[115]
	NF-κB	Mice	[116]
	IL1-β	Rat	[103]
	Leukotrienes, IL-6, MMPs		[120]
Ganglion cell	Par2	Mice	[128]
	GPR91	Rat	[137]

Vascular endothelial growth factor (VEGF) plays a fundamental role in angiogenesis, and its concentration in the vitreous in patients with DR is significantly increased [53]. Although initially considered as a vascular permeability factor, VEGF is recognized as an important contributor to the progression of DR. VEGF also promotes the expression of intercellular cell adhesion molecule-1 (ICAM-1) and initiates early diabetic retinal leukocyte adhesion [54, 55]. Leukocyte adhesion to the vascular endothelium is a necessary first step in DR, mediated by chemokines and adhesion molecules including monocyte chemoattractant protein-1 (MCP-1), chemokine (C-C motif) ligand 2 (CCL2), ICAM-1, and vascular cell adhesion molecule-1 (VCAM-1) [51, 56, 57]. The levels of ICAM-1, VCAM-1, and E-selectin in vitreous are significantly higher in eyes with PDR [58]. ICAM-1 and VCAM-1 are also upregulated in the conjunctiva of diabetic patients with or without retinopathy [51]. TNF-α, together with diabetic duration, remains a single, persistent, independent, and determinant inflammatory marker for PDR [59]. The levels of IL-6, IL-8, IL-5, and IL-10 in the vitreous of patients with PDR are also increased [60]. It appears that inflammatory mediators and adhesion molecules dominate the pathogenesis of DR. However, other mediators that closely related to inflammatory mediator may also be important in the pathophysiology of the disorder. Nuclear factor kappa B (NF-κB) activation was observed in epiretinal membranes of patients with PDR, and selective inhibition of NF-κB reduces the expression of ICAM-1 and VEGF in vivo [61, 62]. Metalloproteinase (MMP)-9 in vitreous is elevated in diabetic patients with retinopathy [63]. Moreover, the level of high-mobility group box-1 (HMGB1) is increased in epiretinal membranes and vitreous fluid from patients with PDR and in the diabetic retina, suggesting HMGB1 is involved in inflammatory and angiogenic signaling pathways in diabetic retina through its putative receptor termed receptor for advanced glycation end products (RAGE) [64].

## The effect of inflammation on diabetic neurosensory retina

### Retina glial cells in neuroinflammation

Retinal glia are classified into two groups: macroglia (Müller cells and astrocytes) and microglia. Each glial subtype differs markedly in distribution, morphology, and pathophysiology. Some studies reported that glial fibrillary acidic protein (GFAP), a glial cell marker, is a sensitive indicator of CNS injury. GFAP is increased in glial cells in patients after 1 to 3 months of uncontrolled diabetes, with pathological potential reduction after longer durations of the disease [65, 66]. Pathological changes in retina glial cells in DR are shown in Table 3.

**Table 3:** Changes of retina glial cells and neurons in DR

Neural cell	Pathology	Cell density	Marker	Function	Signal pathway	Model	References
Müller cell	Nuclear chromatin dispersion, nuclear granulation electrondense	↑	GFAP↑	Proinflammatory	iNOS/COX2	Rat/rMC-cells	[19, 70, 71, 74, 79–81, 84]
				Angiogenic	PEDF	Rat	
					Caspase-1/IL-1β	Rat	
					Wnt/β-catenin	Mice	
					p38 MAPK/NF-κB/IL-6	Human Müller cells	
Astrocyte	Axonal bundles are scanty, starlike cell bodies are irregularly distributed	↓	GFAP↓	Anti-angiogenic	COX-2/EP3/PGE2	Human	[70, 102]
Microglia	Cell bodies appear larger and bore long blunt ruffles with thin thread-like projections	↑	CD45, CD68, HLA-DR	Proinflammatory	MAPK	Rat	[107, 115, 116, 121]
				Angiogenesis	P2 receptors/Ca2+	Rat	
					NF-κB/TNF-α, IL1-β	Mice/rat	
Ganglion cell	Axonal swellings and associated constriction enlarged cell bodies, increased dendritic branches and terminals	↓	Thy1	Neurodegeneration	ERK1/2/COX-2/PGE2	Rat	[129, 136, 137]
					MAPK	Mice	
					NF-κB	Mice	

#### Müller cells

Müller cells are the major glial cell type in mammalian retina, which span the entire depth of the neural retina. Müller cell somata are located in the inner nuclear layer (INL), from which two major trunks extend in opposite directions. The outer trunk forms a network of adherent junctions known as the outer limiting membrane between Müller cells and photoreceptors [67]. In vascularized retina, the end feet contact and surround blood vessels within the retina. The secondary processes branching from the main trunk of Müller cells form extensive sheaths that surround neuronal cell bodies, dendrites, and the axons of ganglion cells [68]. In the normal retina, Müller cells limit the spread of excitatory neurotransmitters such as glutamate, provide metabolic support for a subset of inner retinal cells, and maintain the stability of the extracellular environment [68, 69]. In diseases, Müller cells possess a marked capacity to respond to a wide variety of environmental insults with pathophysiologic and biosynthetic changes [69].

The density of Müller cells is significantly increased at 4 weeks of diabetic rats. The expression of GFAP in Müller cells is not detectable at 4 weeks (early stage) but the expression becomes prominent at 12 weeks. It is noteworthy that hyperplasia of Müller cells precedes GFAP overexpression in the diabetic retina [70]. On electron microscopy, Müller cells in diabetic rats exhibit dispersion of nuclear chromatin and electrondense nuclear granulations, with the presence of increased glycogen, dense bodies, and lysosomes in the cytoplasm [71]. Diabetic retina shows edematous Müller cell end feet in the nerve fiber layer, ganglion cell loss, intercellular space increase in the inner and outer nuclear layers, and outer retina degeneration due to apoptotic cell death as a result of overexpression of caspase-3 [72]. Müller cells are major sources of inflammatory mediators [73] and become “activated” or “reactive” in response to virtually all pathological changes in the retina [74]. By using high-throughput techniques, diabetes-induced alteration of gene expression profile in Müller cells reveals that among 78 altered genes, one third are associated with inflammation [75], suggesting that Müller cells contribute to inflammatory responses during the development of DR. VEGF is rapidly released from Müller cells in early DR, enhancing perfusion by locally increased permeability of blood vessels with concomitant decrease in anti-angiogenic pigment epithelium-derived factor [76, 77]. In VEGF knockout mice, diabetes-induced retinal inflammation, vascular leakage, and vascular degeneration exhibit a significant reduction [76]. In Müller cells cultured in high glucose, the levels of histone acetylation at histone H3 (AcH3K9), AcH3K18, AcH2BK5, and AcH4K8 are increased, with upregulated mRNA of inflammatory genes, such as VEGFR1, IL1-β, ICAM-1, TNF-α, and MCP-1 (CCL2) [78]. These findings suggest that elevation of histone acetylations in Müller cells plays an important regulating role in the inflammatory response under diabetic conditions. The expression of VEGF and pigment epithelium-derived factor (PEDF) in Müller cells is disregulated in high glucose concentration, which contributes to retinal neovascularization in DR [79]. The anti-angiogenic P60, a PEDF derivative, reduces vascular leakage by increasing tight junction proteins in retina vessels through Müller glia signaling and by reducing the levels of inflammatory cytokines that promote vessel abnormalities. The neuroprotective P78, another PEDF derivative, is more effective in the prevention of cell dropout and inner plexiform layer (IPL) thinning with reduction of vitreous levels of TNF-α and IL-2 and activation of the PI3K/AKT pathway in Müller glia [80].

In an STZ-induced diabetic mouse model, disruption of β-catenin in Müller cells attenuates the overexpression of inflammatory cytokines and ameliorates pericyte dropout in the retina. Thus, Müller cell-derived β-catenin is an important contributor to retinal inflammation in DR, and the Wnt/β-catenin pathway is activated in DR model mice [81].

Müller cells produce IL-1 and exert an inhibitory activity on Ag- and IL-2-driven proliferation of T helper cell lines. Under conditions where the inhibitory capacity of Müller cells is suppressed, the cells display APC function to show a dual effect on autoimmune T helper lymphocytes [82]. Müller cells have been reported to produce increased amount of IL-1β when exposed to high glucose in vitro [83], in which caspase-1/IL-1β signaling plays an important role in diabetes-induced retinal pathology [19]. IL-1β has also been reported to induce IL-6 production by Müller cells predominantly through the activation of p38 MAPK/NF-κB signaling pathway [74].

Studies of our laboratory have shown that hyperglycemia induced the overexpression and activation of HMGB1 in Müller cells. HMGB1 mediates toll-like receptor 4 (TLR4)-dependent angiogenesis [84]. The expression of TLR4 was markedly increased in fibrovascular membranes from DR patients and in retinal vascular endothelial cells of diabetic mice [85]. We therefore speculate that Müller cells are involved in inflammation-driven angiogenesis.

Retinal Müller cells (rMC-1) cultured in high glucose increase their production of nitric oxide (NO) and prostaglandin E2 (PGE2) as well as the expression of inducible nitric oxide synthase (iNOS) and cyclooxygenase (COX)-2. In vitro results suggest that hyperglycemia-induced increase in NO in retinal Müller cells promotes the production of cytotoxic prostaglandins via COX-2. iNOS appears to account for the increased production of NO by Müller cells [86].

Exposure of Müller cells to high glucose also induces their expression of RAGE and S100B. RAGE signaling via MAPK pathway was linked to cytokine production. Blockade of RAGE prevents cytokine production induced by high glucose and S100B in Müller cells [87].

Müller cells regulate the level of substances in the neuronal microenvironment. One of the most characterized functions of Müller cells is the regulation of K^+^ in the retina [88]. The accumulation of K^+^ in extracellular space leads to changes in neuronal excitability. Müller cells may also control neuronal activity more directly. When sufficiently depolarized, glutamate uptake by salamander Müller cells is reversed and glutamate is released into extracellular space [89]. Additionally, glycogen stores in the retina are restricted to Müller cells. Furthermore, Müller cells also regulate blood flow in retinal vessels in response to the changes in neuronal activity.

#### Astrocytes

Astrocytes are the primary glia in the brain, constituting approximately one third of the brain mass [90]. Astrocytes in the retina show a stellate morphology, with somata located in the ganglion cell layer and nerve fiber layer (NFL). In the monkey retina, GFAP-positive astrocytes are found ubiquitously in the NFL. Astrocytes are absent in avascular foveal region. The concurrence of retinal astrocytes and intraretinal vascularization may be a common feature for many mammalian species [91]. Despite the fact that astrocytes are far less pervasive in the retina than in the brain, these cells play an important role in the development and maintenance of retinal neurons and blood vessels. They provide energy substrates to neurons and regulate the production of trophic factors and antioxidants in retinal microenvironment [92].

Astrocytes show opposite reactions as compared with Müller cells in response to hyperglycemia. The density of Müller cells is increased, whereas the number of astrocytes is decreased in diabetic retinas. In 4-week diabetic rat retina, astrocyte density is significantly reduced in the peripapillary region and in the far periphery [70]. Astrocytic profiles, notably the processes investing axonal bundles, are scanty in rat diabetic tissue, and the starlike cell bodies are irregularly distributed [70]. In addition, recent study demonstrates that exosomes from retinal astrocytes contain multiple anti-angiogenic components that inhibit laser-induced choroidal neovascularization in model mice [93].

Astrocytes are the major cell population in the optic nerve head and are responsible for the remodeling of the lamina cribrosa structure [94]. Astrocytes are important in stress over-activation of inflammatory responses in glaucoma that leads to local axonal damage within the optic nerve head [95]. Astrocytes have the potential to secrete a wide array of mediators [96]. COX-2 can be constitutively produced by astrocytes and is generally considered as an “immediate early response gene” following damage to the CNS [97]. As an acute phase gene, COX-2 is readily induced in a variety of cells by inflammatory and mitogenic stimuli, including cytokines and growth factors [98]. Overexpression of transforming growth factor-alpha (TGF-α) and epidermal growth factor receptor (EGFR) occurs in active astrocytes [99]. EGFR-dependent induction of COX-2 occurs early in astrocytes following optic nerve injury [100]. COX-2 and COX-2-induced PGE2 participate in DR and regulate the expression of VEGF [101]. In human diabetic retina, COX-2 is induced in astrocytes and contributes markedly to preretinal neovascularization in ischemic retinopathies. This effect appears to be PGE2-mediated mostly via prostaglandin E receptor 3 (EP3) implicating a new interaction through thrombospondin-1 (TSP-1) and CD36 [102].

IL-1β induces its own synthesis in the retinal vascular endothelial cells, Müller cells, and astrocytes. The combination of high glucose stimulation and the upregulation of IL-1β in the diabetic retina is responsible for sustained IL-1β overexpression in astrocytes [103].

#### Microglia

Microglia are bone marrow-derived mononuclear phagocytes, representing the major component of the innate immune cells in the retina [104]. Like macrophages in the rest of the body, microglia use phagocytic and cytotoxic mechanisms to destroy foreign materials. However, microglia differ from macrophages in that they are much more tightly regulated spatially and temporally to maintain proper immune responses in the eye. The size of microglia is small relative to macroglia (such as astrocytes), with changing shapes and oblong nuclei. Microglia together with invading choroidal macrophages significantly contribute to chronic para-inflammation present in several aging retinal pathologies [105].

##### Microglia in human diabetic retinopathy

Microglia settle into the plexiform layers of the retina and gain a highly branched morphology with small cell bodies and long protrusions that may span the complete nuclear layers [106]. In human DR, perivascular microglia in the background form are moderately increased in numbers and are hypertrophic in the inner retinal layers, extending from internal to middle limiting membranes. Hypertrophic microglia in the preproliferative form cluster around cotton-wool spots and infiltrate into optic nerve region. Dilated new vessels in proliferative retinopathy are heavily surrounded by microglia, featuring microglial perivasculitis [107].

##### Microglia in animal models of diabetic retinopathy

Retinal microglia are activated, and the morphology is changed at 4–8 weeks of animal diabetic models [70, 108, 109]. The number of microglia is increased in the outer plexiform layer at 4-month diabetic models. Reactive microglia at 14 to 16-month diabetic models are detected in the outer nuclear and photoreceptor layer [110]. Active Iba1-positive microglia with retracted and swollen processes are present in insulin-2 Akita (Ins2Akita/t) mice after 8 weeks of hyperglycemia [111].

Minocycline, an antibiotic that inhibits microglia, decreases diabetes-induced inflammatory cytokine production and reduces the release of cytotoxins from activated microglia as well as the activity of caspase-3 in rodent retina [112]. Therefore, activated microglia are considered as a major source of proinflammatory and neurotoxic mediators. These cells are also recognized as a potential culprit contributing to the early inflammatory outcome in DR [107, 113].

Advanced glycation end products (AGEs) may act directly on microglia to initiate DR and promote its advancement. AGEs increases the expression of TNF-α in cultured rat retinal microglia, thereby trigging infiltration of leukocytes to the site of vascular injury and causing vascular inflammation [114]. Increased levels of AGEs also lead to the formation of reactive oxygen species (ROS) and ERK/P38 activation during microglial activation in diabetes [109, 115]. Inhibition of the production of NO and other free radicals by glial cells with intracellularly acting antioxidants may imply their ability to reduce AGE-induced neuroinflammatory processes [114]. Identification of the redox-active signal transduction pathways involved in microglial activation and the chemical structures of the responsible AGEs and AGE receptors/binding proteins will provide additional molecular targets for the treatment of AGE-associated inflammatory conditions [114].

NF-κB is activated in pericytes, vascular endothelial cells, macrophages, and microglia in hypoxia-induced C57BL/6N mouse model of neovascularization [116]. NF-κB activation is required for retinal angiogenesis and inhibition of NF-κB ameliorates neuronal cell death in PDR [116]. It is well known that IL-1, IL-6, IFN-γ, and TNF-α activate microglia in vitro [117]. STZ induces a rapid and sustained increase in glycemia and causes microglial activation along with increased levels of TNF-α and IL1-β during a very short period of time [118]. In STZ-induced diabetic rats, TNF-α colocalizes with ionized calcium binding adaptor molecule-1 (Iba-1+) in microglia but not in Müller cells or astrocytes. TNF-α production induced by glycated albumin was blocked by ERK and p38 MAPK inhibitors [119]. Molecules released by activated retinal microglia include glutamate, proteases, leukotrienes, IL-1β, IL-3, IL-6, TNF-α, VEGF, lymphotoxin, macrophage inflammatory protein 1 (MIP-1), and MMPs [120]. Purinergic P2 receptors in high glucose-cultured rat microglia are upregulated, eliciting calcium influx and release of proinflammatory mediators [121]. Furthermore, in Ins2^Akita/t^ mice, a PEDF peptide PEDF78-121 (P78) is effective in preventing cell dropout and IPL thinning, presumably due to its inhibition of microglia activation. P78 also inhibits the activation of PI3K/AKT pathway in Müller glia and reduces vitreous levels of TNF-α and IL-2 in vitro [80].

### Neurons in neuroinflammation

Neurons in retina include photoreceptors, bipolar cells, amacrine cells, and retinal ganglion cells (RGCs). Diabetes affects both neurites (axons and dendrites) and cell bodies of retinal neurons, as evidenced by neuritic swellings [122].

#### RGCs

Diabetes impairs axonal retrograde transport in large- and medium-sized RGCs in type 1 but not type 2 diabetic rats [123]. The total number of RGC bodies is reduced in type 2 diabetic rats [14]. There is a reduction in the overall thickness of inner layers of the retina, accompanied by diminished number of RGCs in rat retinas after long term experimental diabetes [124]. After 22 weeks of hyperglycemia, there is a 23.4 % reduction in the number of cell bodies in the RGC layer in Ins2Akita mice [125]. In diabetic patients, the number of RGCs is also reduced [124, 126]. Consistent with this, scanning laser polarimetry revealed a reduced thickness of the nerve fiber layer in diabetic patients [127]. The morphology of a subset of RGCs is altered in diabetes, including axonal swellings with associated constriction, enlarged cell bodies, and increased dendritic branches and terminals [127].

The proteinase-activated receptor-2 (Par2) is recognized for its marked proangiogenic properties in the retina [128]. Par2 mRNA in cultured retinal neuronal cells (RGC-5) is increased by IL-1β [129]. Par2 stimulation activates several downstream effector events, including Ca2+ mobilization and MAPK [130, 131]. RGC-5 cells treated with SLIGRL exhibit increased MAPK signaling, including Erk1/2, Jnk, and p38 phosphorylation [129].

Regeneration of injured RGCs is supported by Müller cell-derived neurotrophic/protective factors [132]; among those are VEGF [133], ciliary neurotrophic factor (CNTF) [134], and PEDF [135]. PEDF activates NF-κB in RGC. Addition of NF-κB inhibitor (SN50) to PEDF-treated RGC reduces their survival. Thus, NF-κB activation in RGC is critically involved in the effect of Müller cell-derived PEDF on maintaining neuronal survival [136].

Recent research demonstrates that hyperglycemia causes succinate accumulation and G protein-coupled receptor 91 (GPR91) activation in RGC, which mediate VEGF-induced retinal vascular change via the ERK1/2/COX-2/PGE2 pathway [137]. Proinflammatory and proapoptotic thioredoxin-interacting protein (TXNIP) has a causative role in the development of diabetes [138, 139]. TXNIP expression is increased in the brain of diabetic rats [140] and plays a role in RGC injury in glaucoma [141, 142]. Blocking the expression of TXNIP in diabetic rat retinas results in the inhibition of its target genes COX-2 and FN thus demonstrating TXNIP’s role in aberrant gene induction in early DR. RNAi silencing TGS of TXNIP abolishes diabetes-induced retinal gliosis and ganglion injury [143].

#### Other neurons

Amacrine cells are the third-order retinal interneurons, projecting their processes into the IPL and contribute to the most of the synapses in the inner plexiform layer and mediate visual information input from bipolar cells onto retinal ganglion cells [144]. Mammalian AII retinal amacrine cells are arrow-field, multistratified glycinergic neurons best known for its capacity to collect scotopic signals from rod bipolar cells and distribute the signals to ON and OFF cone pathways across the network [145].

There are three classes of photoreceptors: rods, long-wave system (LWS) cones, and short-wave system 1 (SWS1) cones. Each class displays a distinct morphology as well as visual pigment. Because photoreceptors are especially vulnerable to hypoxia [146], diabetes may also affect the function of photoreceptors. It has been reported that there are foveal cone photopigment bleaching abnormalities in patients with diabetes [147]. There are decreases in the sensitivity parameter (log S) for both rod-isolated and cone-isolated ERG a-wave responses in patients with DR. Moreover, rod and cone b-wave changes in DR patients, including changes in both amplitude and implicit time [148]. However, the function of these neurons in DR remains unclear.

##### Immuno-inflammatory response in DR

It has recently been recognized that the pathology of diabetic retinopathy has strong immunological underpinnings [149]. T cell abnormalities are believed to be the major cause of autoimmune disease in type 1 diabetes, leading to the destruction of pancreatic islets. In type 2 diabetes, inflammation and activation of monocytes are important for enhancing insulin resistance and may contribute to the loss of insulin secretory function of islet cells [150]. In many diabetic complications, there is dysregulation of innate immunity associated with increased inflammatory responses [151]. Improper activation of the innate immune system may result in DR. TLR4 is an important mediator of innate immunity, and genetic alterations of TLR4 is associated with inflammation in the hyperglycemic condition [152].

Resident microglia are regarded as immunological watchdogs in the brain and retina. These cells are active sensors of neuronal microenvironment and rapidly respond to insults with morphological and functional transformation into reactive phagocytes [153]. Inflammation in diabetes activates microglia, stimulates a cascade of inflammation that recruits leukocytes, causes vascular breakdown, and directly induces glial dysfunction and neuronal cell death through the release of cytotoxic substances. Increased levels of sICAM-1 and sVCAM-1 as well as high concentrations of vitreous IL-6 and TNF-α in patients with PDR appear to confirm the inflammatory-immune nature of PDR [154].

## DR is a result of systemic neuroinflammation

### CNS inflammation in diabetes

Diabetes causes chronic inflammatory complications in the peripheral and CNS. Amylin deposition is promoted by chronic hyperamylinemia, which is common in humans with pre-diabetic insulin resistance. The majority of patients with T2DM have abundant amylin amyloid deposition in the pancreas [155]. A recent study indicates that chronic hyperamylinemia promotes the accumulation of oligomerized amylin in the brain, which may trigger inflammatory responses and lead to neurological defects [156]. β-amyloid deposition around brain microvessels can cause direct toxicity to microvascular endothelial cells (BMVECs). Impaired clearance of β-amyloid across the blood brain barrier (BBB), aberrant angiogenesis, and senescence of the cerebrovascular system may initiate neurovascular uncoupling, brain hypoperfusion, and neurovascular inflammation [157].

T1DM is also associated with increased expression of proinflammatory mediators, such as IL-1β, IL-2, IL-6, TNF-α, and NF-κB, compared to age matched control brains [21, 158]. In addition, TNF-α and IL-1β induce COX-2 activity in perivascular macrophages of BBB and generate prostaglandin E2, which enters the brain and stimulates paraventricular nucleus (PVN) neurons to release adrenocorticotropic hormone (ACTH). Increased expression of Ang II, ICAM-1, lymphocyte function-associated antigen-1 (LFA-1) and CD8 positive cells are found in diverse zones of the cerebrum and cerebellum in STZ-induced diabetic rats [159]. Local Ang II increases vascular permeability by promoting the secretion of VEGF [160]. Ang II also contributes to the recruitment of inflammatory cells into tissues by stimulating the production of cytokines and chemokines.

### Systemic inflammation in diabetes

Connections of neuropathy with the bone marrow (BM), CNS, and peripheral nervous system may exist. Systemic hyperglycemia-induced inflammation in diabetes may result in BM neuropathy by enhancing the generation of inflammatory cells and lead to vascular complications such as DR by reducing the production of endothelial progenitors, which maintain the endothelial function and renewal. [21]. Endothelial progenitor cells (EPCs) arising from BM circulate in the bloodstream and traffic to areas of injury to orchestrate vascular repair [161, 162]. Diabetic individuals have fewer EPCs in the circulation with decreased migratory and reparative potential. Acellular capillaries in the retina in type 2 diabetic rats are observed at the precise time when there is denervation of the BM and reduction in peripheral clock gene expression. The resultant acellular capillaries appear at 4 months of diabetes, due to the loss of proper EPC reparative function and the failure of circadian EPC release secondary to diabetes-associated denervation of BM [163]. Therefore, BM neuropathy precedes the development of DR. The decrease in circulating EPCs reduces the repair of injured retinal vessels in diabetes and leads to the development of acellular capillaries.

BM-derived cells such as leukocytes play a critical role in the development of diabetic retinopathy in animals [164]. Diabetes-induced inflammatory changes, superoxide production, and degeneration of retinal capillaries are inhibited in diabetic mice in which inflammatory proteins (iNOS and PARP-1) are deleted from BM cells [165].

## Intervention of neuroinflammation in DR

Pharmacologic interventions are available to reduce neural inflammatory response in patients with DR [21], in particular in patients who fail to respond to anti-VEGF therapy. Neuroprotection as a new approach to the treatment of early stage DR has been emphasized. Neuroprotection effect is based on administering natural protective factors that may downregulate inflammatory responses in the diabetic retina. The factors such as pigment epithelial growth factor, somatostatin, corstistatin, and neurotrophins are abundant in the physiological retinas. Therefore, administration of these factors could be considered as replacement treatments [166].

### Hesperetin

Hesperetin (3′,5,7-trihydroxy-4-methoxyflavanone) is a member of the flavanone subclass of flavonoids. It is a potential anti-inflammatory agent with potent inhibition of LPS-induced expression of the COX-2 gene in RAW 264.7 macrophage cell line [167]. Hesperetin also inhibits the appearance of oxidative stress biomarkers, such as thiobarbituric acid-reactive substance (TBARS) and carbonyl content. Moreover, hesperetin activates catalase and total superoxide dismutase (SOD) in mice [168]. Thus, hesperetin may be neuroprotective as shown by the fact that hesperetin-treated retina reduces the expression of caspase-3, GFAP, and AQP4, which are increased in diabetic rat retina [72].

### Minocycline and doxycycline

Minocycline (MINO) and doxycycline (DOXY) derived from tetracycline show neuroprotection in animal models of ischemia [169–171]. MINO exerts anti-inflammatory effect on microglia by inhibiting the production of inflammatory mediators, such as NO, cyclooxygenases, prostaglandins, IL-1β, and TNF-α, while DOXY downregulates NO and IL-1β [169, 172–174]. MINO inhibits hyperglycemia-induced histone acetylations, Müller cell activation and upregulation of inflammatory mediators [175]. MINO also inhibits the formation of acellular capillaries in the retina of diabetic and galactosemic mice. The activation of caspase-1 and caspase-3 by high glucose and subsequent neuronal apoptosis in retina Müller cells and microglia are also inhibited by MINO [19, 112, 176]. In the clinic, MINO improves visual acuity and neuropathic pain resulted from inflammation in diabetic patients [21]. These findings support that MINO is a novel promising therapeutic drug for DR [21].

### VEGF

VEGF is an angiogenic and vessel-permeability factor [177]. Anti-VEGF therapy in the management of PDR and DME has shown beneficial effects [178, 179]. Antibodies Ranibizumab (Lucentis®) [180, 181], Bevacizumab (Avastin®) [182, 183], and Aflibercept (Eylea®) [184], which inhibit VEGF isoforms, are currently used in the clinic. Most studies focus on inhibition of vascular permeability and endothelial cell proliferation stimulated by VEGF [185]. However, further research suggests that VEGF also has neurotrophic and neuroprotective activity [186]. The hypoxia-induced neuroprotective effects sequentially require the activation of VEGF/VEGFR-2 and Akt/PKB phosphorylation, indicating that VEGF is a hypoxia-induced neurotrophic factor [187]. VEGF also has a protective effect on hippocampal neurons against glutamate-mediated toxicity, and this effect is dependent on PI3-K/Akt and MEK/ERK signaling pathways mediated primarily through Flk-1 receptor [188].

Therefore, VEGF may have a dual role: neuroprotection and neovascularization in hypoxic regions of the tissues. Its effect on angiogenesis and vascular permeability appears paradoxical versus the neuroprotective activation [14]. Patients who failed in anti-VEGF therapy may be due to inhibition of its neuroprotective function. Moreover, diabetic patients may be at higher risk for both systemic and ocular complications, such as cardiovascular and renal diseases, susceptibility to infection, endophthalmitis, retinal detachment, and intraocular hemorrhage [189–192]. Thus, although anti-VEGF therapy is helpful, complications of this treatment should not be overlooked.

### Other novel therapeutic medicine

There are novel therapies focusing on inflammation and neurodegeneration to mitigate retinal damage associated with diabetes. Cannabidiol (CBD) is a non-psychoactive component considered to have the properties of anti-inflammation and anti-oxidation [193, 194]. CBD attenuates high glucose-induced NF-κB activation in human coronary endothelial cells (HCAECs) in vitro. It also attenuates high glucose-induced iNOS expression and 3-nitrotyrosine (3-NT) formation in endothelial cells [195]. Moreover, CBD decreases the incidence of diabetes possibly through an immunomodulatory mechanism that induces regulatory Th2 responses [196]. It is reported that CBD reduces neurotoxicity, inflammation, and BRB breakdown in STZ-induced diabetic rats by blocking activation of microglia and p38 MAP kinase, a downstream molecule of proinflammatory cytokines and oxidative stress [197]. Thus, CBD is a promising candidate for anti-inflammatory and neuroprotective therapy for DR.

Resveratrol is a natural polyphenol found in grapes and red wine. Resveratrol has protective effects on atherosclerosis and cardiovascular diseases through reduction in oxidative stress [198–200]. Further research demonstrates that resveratrol protects diabetic neuropathy by improving motor nerve conduction velocity and nerve blood flow, as well as reduction in nociception [199]. A recent study indicates that resveratrol inhibits the activation of NF-κB and TNF-α and reduces apoptotic cells in the retina of type 2 diabetic rats [201]. Resveratrol also exerts its neuroprotective effect on RGCs by activating the sirtuin 1 pathway in an optic nerve transection rat model [202].

The non-steroidal anti-inflammatory drugs (NSAIDs) have been used to treat DME by inhibiting prostaglandin biosynthesis [203, 204]. Injection of intravitreal diclofenac (IVD) sodium, as a potent NSAIDs, has been used in the treatment of macular edema of many etiologies such as uveitic CME, diabetic macular edema, and retinal vein occlusions [203]. A randomized double-masked clinical trial demonstrated that the effect of injection of IVD was superior to intravitreal injection of bevacizumab (IVB) in the treatment of naïve DME [205]. Therefore, using IVD as an adjunct or alternative treatment may enhance the functional outcome of naive DME.

## Conclusions

DR manifests characteristics of chronic neuroinflammation. Neurosensory retina including retina glial cells and neurons are involved in neuroinflammatory responses of DR. A caveat that should be kept in mind is that most DR pathogenic studies are conducted on animals, but none of the animal models may replicate all features of human disease possibly due to different anatomic characteristics of retinal structure. Nevertheless, animal models remain necessary tools to study the pathogenesis of diseases and have provided useful information for better understanding of the pathogenesis of neuroinflammation in DR. Many features simulate the process of human disease and may aid the development of more efficient therapeutic strategies against human DR.

## Abbreviations

AGE: Advanced glycation end products
AION: anterior ischemic optic neuropathy
BM: bone marrow
CNS: central nervous system
COX: cyclooxygenase
DME: diabetic macular edema
DOXY: doxycycline
DR: diabetic retinopathy
ERG: electroretinogram
GFAP: glial fibrillary acidic protein
HMGB1: high-mobility group box-1
ICAM-1: intercellular cell adhesion molecule-1
IL: interleukin
IRMAs: intraretinal microvascular anomalies
MINO: minocycline
NF-κB: nuclear factor kappa B
OPs: oscillatory potentials
PION: posterior ischemic optic neuropathy
RGCs: retinal ganglion cells
T2DM: type 2 diabetes mellitus
TNF-α: tumor necrosis factor-alpha
VCAM-1: vascular cell adhesion molecule-1
VEGF: vascular endothelial growth factor
VEPs: visual-evoked potentials
